# Decellularized Intervertebral Discs: A Potential Replacement for Degenerate Human Discs

**DOI:** 10.1089/ten.tec.2020.0104

**Published:** 2020-11-17

**Authors:** Halina T. Norbertczak, Eileen Ingham, Hazel L. Fermor, Ruth K. Wilcox

**Affiliations:** ^1^ Institute of Medical and Biological Engineering, School of Biomedical Sciences, Faculty of Biological Sciences, The University of Leeds, Leeds, United Kingdom.; ^2^ Institute of Medical and Biological Engineering, School of Mechanical Engineering, Faculty of Engineering and Physical Sciences, The University of Leeds, Leeds, United Kingdom.

**Keywords:** extracellular matrix, intervertebral disc, acellular biological matrices

## Abstract

Intervertebral disc (IVD) degeneration is a major cause of back pain. Current surgical interventions have limitations. An alternative approach is to replace degenerated IVDs with a natural biological scaffold. The removal of cellular components from human IVDs should render them nonimmunogenic upon implantation. The aim of this initial proof of technical feasibility study was to develop a decellularization protocol on bovine IVDs with endplates (EPs) and assess protocol performance before application of the protocol to human IVDs with attached EP and vertebral bone (VB). A decellularization protocol based on hypotonic low concentration sodium dodecyl sulfate (0.1% w/v) with proteinase inhibitors, freeze/thaw cycles, and nuclease and sonication treatments was applied to IVDs. Histological, biochemical, and biomechanical comparisons were made between cellular and decellularized tissue. Cell removal from bovine IVDs was demonstrated and total DNA levels of the decellularized inner annulus fibrosus (iAF), outer annulus fibrosus (oAF), and EP were 40.7 (±11.4), 25.9 (±3.8), and 29.3 (±3.1) ng.mg^−1^ dry tissue weight, respectively (*n* = 6, ±95% confidence level [CL]). These values were significantly lower than in cellular tissue. No significant difference in DNA levels between bovine cellular and decellularized nucleus pulposus (NP) was found. Glycosaminoglycans (GAGs) were largely retained in the NP, iAF, and oAF. Cyclic compression testing showed sufficient sensitivity to detect an increase in stiffness of bovine IVD postdecellularization (2957.2 ± 340.8 N.mm^−1^) (predecellularization: 2685.4 ± 263.1 N.mm^−1^; *n* = 5, 95% CL), but the difference was within natural tissue variation. Total DNA levels for all decellularized tissue regions of human IVDs (NP, iAF, oAF, EP, and VB) were below 50 ng.mg^−1^ dry tissue weight (range: 2 ng.mg^−1^, iAF to 29 ng.mg^−1^, VB) and the tissue retained high levels of GAGs. Further studies to assess the biocompatibility and regenerative potential of decellularized human IVDs *in vitro* and *in vivo* are now required; however, proof of technical feasibility has been demonstrated and the retention of bone in the IVD samples would allow incorporation of the tissue into the recipient spine.

**Impact statement:**

Intervertebral disc (IVD) degeneration is a major cause of back pain. Current surgical treatments have limitations and relatively poor outcomes. An implantable cell-free biological scaffold, which will not invoke adverse immune responses, has the potential to preserve the natural mobility of the patient's spine and be regenerated with endogenous cells, preventing further degeneration and improving surgical outcomes. This study demonstrates, for the first time, that it is possible to create a cell-free human IVD biological scaffold with attached bone using decellularization technology, the first step toward the development of an implantable regenerative device for IVD replacement.

## Introduction

Back pain places a huge burden on individuals and economies. In 2010, the global prevalence of lower back pain was estimated to be 9.4% and was ranked as the greatest contributor to disability in North America and Europe.^1^

**FIG. 1.**
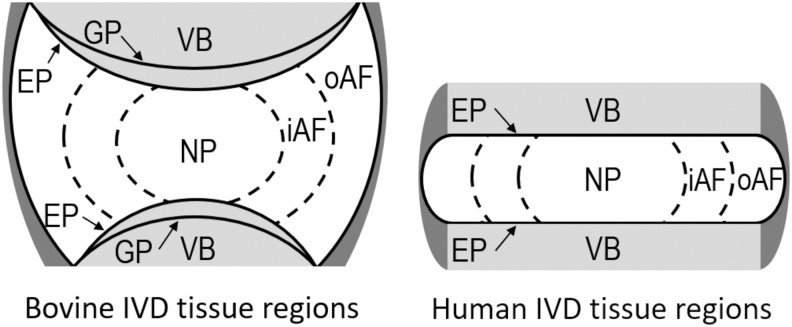
Tissue regions of bovine and human IVDs, showing the NP, iAF, oAF, VB, GP, and EP (comprised the cartilaginous EP and vertebral EP, integrated into the IVD and VB, respectively). EP, endplates; GP, growth plate; iAF, inner annulus fibrosus; IVD, intervertebral disc; NP, nucleus pulposus; oAF, outer annulus fibrosus; VB, vertebral bone.

The degeneration of intervertebral discs (IVDs) is associated with back and referred pain.^2^ IVDs are composed of distinct structures (Fig. 1): a proteoglycan-rich nucleus pulposus (NP) surrounded by the fibrocartilaginous inner and outer annulus fibrosus (iAF and oAF).^2,3^ IVDs are integrated into adjacent vertebral bone (VB) by the endplates (EP), which can be subdivided into the vertebral EP, a layer of cortical bone, and the hyaline cartilaginous EP.^4,5^ The IVD forms an enthesis-like region at the cartilaginous EP. During IVD degeneration, there is a loss of glycosaminoglycans (GAGs) leading to IVD dehydration and reduced mechanical performance. The resulting decrease in IVD height can cause compression of facet joints, nerves, and the spinal cord leading to pain.^2,3^

Current surgical treatments for IVD degeneration (spinal fusion and total disc replacement) have limitations such as adjacent level degeneration and subsidence.^6^ Decellularization of natural biological tissues aims to remove cellular components, while retaining the collagen -rich extracellular matrix (ECM), histoarchitecture, and functional molecules.^7^ The decellularized tissue can be used as a scaffold to surgically replace diseased and damaged tissues.^7,8^ Free of cells and DNA, the scaffold should not invoke an adverse immune response in the host. The ECM comprised components that are highly conserved between mammalian species, allowing scaffolds derived from an allogeneic or xenogeneic source to be tolerated by the host.^9,10^

Particularly important for the replacement of musculoskeletal tissues is the retention of the mechanical properties postdecellularization to allow for immediate restoration of function before endogenous cells undergo constructive remodeling of the scaffold.^8^ It is proposed that IVDs can be decellularized without compromising biomechanical function.

There have been limited investigations into decellularization of the IVD, with the majority of studies focusing on individual animal IVD tissue regions (NP and AF) rather than intact IVDs.^11–16^ Booth *et al.* originally reported on the decellularization of cardiovascular tissues utilizing low concentration sodium dodecyl sulfate (SDS) with protease inhibitors.^17^ An adaptation of this protocol was applied to porcine menisci and, despite a reduction in GAGs, there were no significant changes in compressive and tensile properties of the tissue.^18^ Menisci are similar to IVDs (fibrocartilaginous and integrated into adjacent bone forming enthesis-like structures), which suggested that a similar approach would be successful on whole IVDs. Moreover, nondecellularized cervical IVD allografts with EP attachments have shown promising results up to 10 year follow-up, demonstrating that the surgical procedure is achievable.^19,20^

It is therefore proposed that IVDs can be decellularized with a view to future development of implants to replace degenerate IVDs. The aims of this initial proof of technical feasibility study were to assess the performance of a decellularization protocol on a large animal model (bovine tail IVDs with EPs) before application of the protocol to human IVDs with attached VB.

## Methods

### Tissue dissection

#### Bovine tails

(Limousin cattle, mixed gender, ∼28 months old) were received from an abattoir within 5 hours of slaughter. The soft tissues and processes were removed from the caudal vertebrae. The two largest IVDs were extracted with ∼5 mm of VB to each side. The growth plates were exposed and removed using an electrical burr, leaving 1-2 mm of VB above each EP (EP-IVD-EP specimens). The largest (C1-C2) specimens were decellularized and the second largest (C2-C3) specimens served as matched untreated cellular controls; adjacent bovine tail IVDs do not differ significantly in GAG and DNA content.^21^

#### Human donor tissue

Following ethics approval (Yorkshire and The Humber Health Research Authority and Sheffield Research Ethics Committee REC ref 15/YH/0096), whole donor spines were obtained from the Leeds GIFT Research Tissue Project, St. James' Hospital, Leeds, UK (*n* = 3, male 38 and 75 years, female 54 years). Spines were thawed from −80°C to 4°C. The lumbar and lower thoracic regions were separated from the upper thoracic spine by cutting through the transverse plane of the T7 vertebrae. The ribs and spinous processes were removed from the upper thoracic spine and the IVDs were extracted by cutting midway through the transverse plane of each vertebra.

The resulting VB-IVD-VB specimens were cleaned of soft tissues and scanned in a μCT scanner (μCT100; Scanco Medical AG, Brüttisellen, Switzerland). Sagittal and coronal plane, low-resolution 2D scout view images were used to select study samples with approximately equal VB thickness on the cranial and distal surfaces; equal VB depth across the sample; and with no evidence of IVD collapse or damage. Two specimens from each spine (T1-T2 and T5–T6) were decellularized. Adjacent specimens (T2–T3 and T6–T7) served as matched untreated cellular controls.

### Decellularization protocol

Specimens underwent three freeze-thaw cycles between −20°C and room temperature. Bony surfaces were irrigated with Dulbecco's phosphate-buffered saline (DPBS, Oxoid; pH 7.2) at 37°C, using a dental flosser (Ultra Water Flosser WP-120, Waterpik, UK). Specimens underwent further three freeze-thaw cycles in hypotonic buffer (10 mM tris, Sigma-Aldrich, and 10 KIU.mL^−1^ aprotinin, Trasylol^®^, Bayer; pH 8) and were decontaminated in Cambridge antibiotic solution (Source BioScience) at 37°C with horizontal agitation on an orbital shaker (PSU-10I, Grant bio) at 80 rpm for 1 hour.

Specimens were cycled between hypotonic buffer and hypotonic buffer containing 0.1% w/v SDS (Sigma-Aldrich) for 14 days with solution changes every 24 or 72 hours (for weekend incubations) at alternating temperatures of 4 and 42°C, 240 rpm. Samples were washed in DPBS containing 10 KIU.mL^−1^ aprotinin for 4 days (with changes of fresh solution) at 42°C, 240 rpm. Samples were incubated in nuclease solution (50 mM tris, Sigma-Aldrich; 1 mM magnesium chloride, VWR International; and 10 U.mL^−1^ Benzonase, Novagen; pH 7.5) at 37°C, 80 rpm, for 3 hours, for two cycles and then one 16-hour cycle. Samples were washed in DPBS followed by hypertonic buffer (50 mM tris; 1.5 M sodium chloride, ThermoFisher Scientific; pH 7.5) and again in DPBS (all at 42°C, 24 hours, 240 rpm).

Surface decontamination of the specimens was carried out using 0.1% v/v peracetic acid (PAA, Sigma-Aldrich; pH 7.2) at 27°C, 160 rpm, for 3 hours. Samples were washed in DPBS for 14 days (solution changes every 24 or 72 hours) at alternating temperatures of 4°C and 42°C, 160 rpm. At the end of all incubation steps, except nuclease and PAA treatments, samples were subject to ultrasonication (44 kHz) for 10 minutes. All solutions were sterile and changes were made aseptically. Solution volume was 300 mL, except for Cambridge antibiotic solution where 20 mL was required to cover each sample.

## Experiment

### Experimental design

#### Decellularization

Bovine specimens were dissected and decellularized according to the protocols above. Protocol performance was assessed through histological, biochemical, and biomechanical evaluation of decellularized tissue that was compared to untreated cellular controls. The decellularization protocol was then applied to human specimens, which were evaluated histologically and biochemically.

#### Histological evaluation

For bovine EP-IVD-EP specimens, five-millimeter-thick sagittal slices were taken from the center of cellular and decellularized samples (*n* = 6). Two cellular and two decellularized VB-IVD-VB specimens from each human donor (*n* = 3) were dissected as above through the coronal plane. Slices were fixed for 48 hours in 10% (v/v) neutral buffered formalin (Atom Scientific), demineralized in 12.5% (w/v) ethylenediaminetetraacetic acid (EDTA; Fisher Scientific; pH 7) at 42°C and 240 rpm for 7 days, and fixed again (48 hours).

Samples were processed automatically (Leica TP1020 tissue processor, Leica Biosystems) and embedded in paraffin wax. Histological sections (6 μM) were stained with Mayer's hematoxylin and eosin Y, (H&E; Atom Scientific and Merck Millipore respectively); 4′, 6-diamidino-2-phenylindole (DAPI; Sigma-Aldrich), or Safranin O and Fast Green (Acros Organics and Sigma-Aldrich, respectively). Safranin O staining intensity was qualitatively assessed. One representative field of view (100 × magnification) from a histological section from each sample was graded using a scale of 0 (no staining) to 5 (intense staining). This was carried out for each tissue region (NP, iAF, oAF, EP, and VB) within each sample. Mean staining intensity scores were calculated.

#### Biochemical evaluation

Tissue not used for histological evaluation, described above, was divided into tissue regions (NP, iAF, oAF, EP, and VB). NP, iAF, and oAF samples were macerated with a scalpel blade. EP and VB samples were crushed using a pestle and mortar. Samples (*n* = 6 bovine; 2 × *n* = 3 human) were lyophilized to constant weight before biochemical evaluation:

##### DNA quantification

Total DNA was extracted from known weights of lyophilized tissue samples using the DNeasy Blood and Tissue kit (Qiagen), according to the manufacturer's instructions. For the EP and VB, an in-house digestion buffer (12.5% [w/v] EDTA and 1% [w/v] SDS) was used with the provided proteinase K enzyme (600 mAU/mL) in place of the kit buffer. Extracted DNA was quantified at an absorbance of 260 nm (Nanodrop ND-1000; Labtech International) and expressed in ng.mg^−1^ of dry tissue weight.

##### GAG quantification

Known weights of lyophilized NP, iAF, and oAF samples were digested in papain solution (5 mM L-cystine hydrochloride, Sigma-Aldrich; 5 mM EDTA; and 800 kU.mL^−1^ papain, Applichem; pH 6) at 60°C for 48 hours. Sulfated GAG content was quantified according to Farndale *et al.*^22^ Briefly: chondroitin sulfate (Sigma-Aldrich) standards and test samples (40 μL) were added to flat-bottomed 96-well plates with 250 μL 1,9 dimethylmethylene blue (DMMB; Sigma-Aldrich). After 2 minutes, the absorbance at 525 nm was measured. The GAG concentration of the test samples was interpolated from the linear region of the standard curve, taking in to account the dilution factor, and expressed in μg.mg^−1^ dry weight.

#### Biomechanical testing of bovine IVDs

Bovine C1-C2 EP-IVD-EP specimens (*n* = 5) were biomechanically tested before and after decellularization. Specimens were cemented into polymethyl methacrylate (PMMA, Centribase, WHW Plastics Ltd.) end caps with flat and roughly parallel surfaces, compatible in size with the custom fixtures of the material testing machine.

##### Hydration

Cemented specimens were placed into a custom-made multistation static compression rig, submerged in 37°C DPBS. A 40 N load was applied for 24 hours to ensure all specimens were in a similarly hydrated state before testing. The load was chosen to exert an approximate nuclear pressure of 0.1 MPa, reported to be equivalent to the lowest physiological pressures experienced in human IVDs.^23–25^ Individually, specimens were transferred to a material testing machine (ElectroPuls E10000; Instron Ltd., UK) fitted with a 10 kN load cell and placed between testing platens submerged in 37°C DPBS. The upper platen was attached to two stacked linear bearings, allowing movement of the upper fixture in the horizontal plane, which prevented over constraint of the sample.

##### Cyclic compression

Following a 30-minute static hold, applied to exert a nuclear pressure of 0.1 MPa, cyclic loading (100 load-unload cycles, 1 Hz between 383 and 800 N) was applied to generate estimated nucleus pressures corresponding to the extremes experienced by human IVDs during daily activities.^23–25^

##### Data processing

Load and displacement data were extracted from the material testing machine and the specimen stiffness over each load cycle was determined using in-house scripts (Python).

#### Data analysis

Statistical analysis was carried out using IBM SPSS Statistics 26. Due to low specimen numbers, normal distribution of the data could not be assessed and so nonparametric statistical tests were carried out: Mann–Whitney *U* test for unpaired data and Wilcoxon signed rank test for paired data. A *p*-value <0.05 was accepted as significant.

### Experimental results

#### Histological evaluation of bovine specimens

**FIG. 2.**
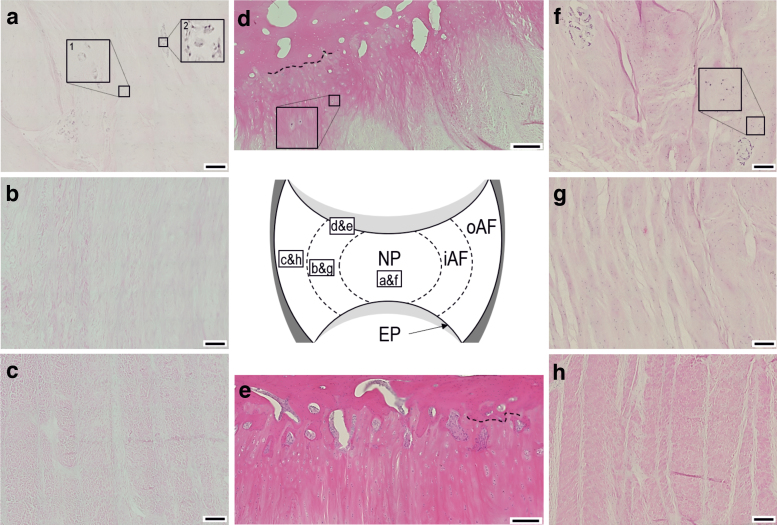
Images of H&E-stained tissue sections of bovine IVD tissue regions. Decellularized NP **(a)**; iAF **(b)**; oAF **(c);** and EP **(d)**. Cellular EP **(e)**; NP **(f)**; iAF **(g);** and oAF **(h)**. The central diagrammatic representation of a bovine IVD cross-section shows the location of the tissue regions shown in the individual images (*boxed areas* with image labels). Images a, d, and f include magnified *inset images* of selected regions. The *dashed lines* in images d and e highlight the interface between the IVD enthesis and EP. All images taken at × 200 magnification with 200 μM scale bars. H&E, hematoxylin and eosin. Color images are available online.

**FIG. 3.**
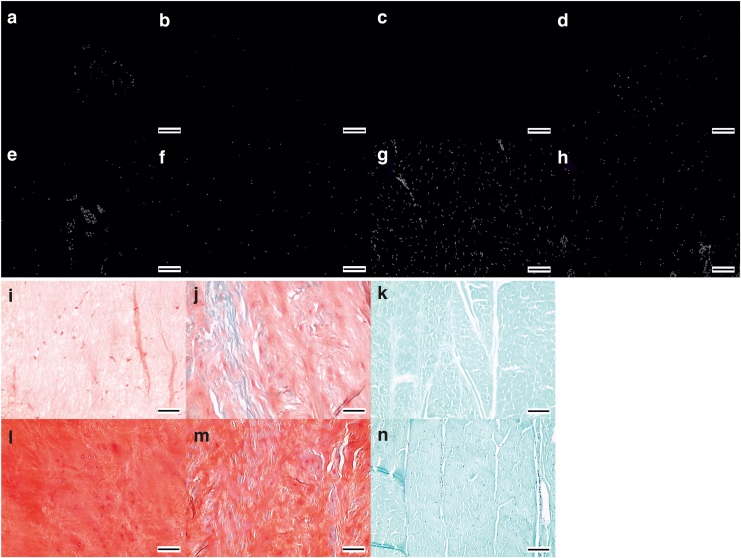
Images of histologically stained tissue sections of bovine IVD tissue regions. DAPI-stained sections of decellularized NP **(a)**; iAF **(b)**; oAF **(c);** and EP **(d)** and cellular NP **(e)**; iAF **(f)**; oAF **(g);** and EP **(h)**. Safranin O/fast green stained sections of decellularized NP **(i)**; iAF **(j);** and oAF **(k)** and cellular NP **(l)**; iAF **(m)** and oAF **(n)**. All images taken at × 100 magnification with 100 μM scale bars. DAPI, 4′, 6-diamidino-2-phenylindole. Color images are available online.

Decellularized tissue was largely devoid of cells. H&E- and DAPI-stained sections showed a reduction in nuclear material for all decellularized tissue regions: NP, iAF, oAF, and EP (Figs. 2a–d and 3a–d), compared to cellular tissue regions (Figs. 2e–h and 3e–h). Nuclei in cellular tissue sections showed clearly defined edges and intense staining with hematoxylin and DAPI stains (Figs. 2f inset and 3e). NP cells were either single or present in high-density clusters (Figs. 2f and 3e). *Single* clearly defined nuclei were not observed in the decellularized NP, although some “ghost nuclei” were present as diffuse and weakly stained areas (Fig. 2a, inset 1), suggesting a reduction in nuclear material. Cell nuclei in the decellularized NP cell clusters appeared fragmented and diffusely stained with occasional more intensely stained areas resembling intact nuclei (Figs. 2a, inset 2 and 3a).

Intact and ghost nuclei were rare in sections of the decellularized iAF and oAF (Fig. 2b, c). Cell nuclei were largely removed from the enthesis regions between the IVD and EP; when present, they were at the IVD side of the interface (Figs. 2d, inset and 3d). GAGs were retained in the decellularized IVDs. Safranin O staining of decellularized NP and iAF sections (Fig. 3i, 3j) was at a lower intensity than for cellular tissue (Fig. 3l, m). The oAF did not show any Safranin O staining predecellularization and postdecellularization (Fig. 2k, n). Mean (*n* = 6) staining intensity scores of cellular NP, iAF, and oAF were 4.6, 4.5, and 0, respectively. Mean (*n* = 6) staining intensity scores of decellularized NP, iAF, and oAF were 2.5, 3, and 0, respectively.

The histoarchitecture of decellularized bovine tissue resembled that of cellular tissue. The ECM of the decellularized NP (Fig. 3i) retained a random fiber alignment that was slightly looser compared with cellular NP (Fig. 3l). The iAF and oAF retained their parallel lamella structure (Fig. 3j, k, m and n). No evidence of gross changes to the histoarchitecture of the EP was observed in decellularized tissue sections (Fig. 2d) compared to cellular samples (Fig. 2e).

#### Biochemical evaluation of bovine specimens

##### DNA

**FIG. 4.**
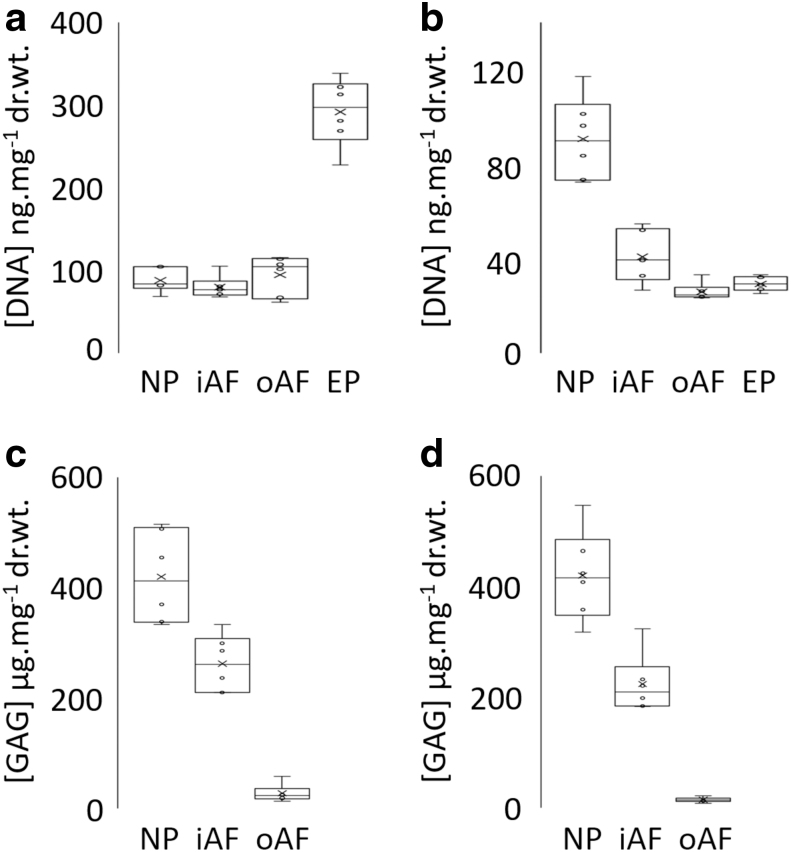
Mean (*n* = 6) total DNA content (ng.mg^−1^ dry tissue weight) of cellular **(a)** and decellularized **(b)** human IVD tissue regions. Mean (*n* = 6) sulfated GAG content (μg.mg^−1^ dry tissue weight) of cellular **(c)** and decellularized **(d)** human IVD tissue regions. x = mean; ○ = individual data points; mid-line of box = median; Bars = extreme values; Box = interquartile range. GAG, glycosaminoglycan.

There was a reduction in the mean total DNA content in decellularized oAF, iAF, and EP (40.7 ± 11.4, 25.9 ± 3.8, and 29.3 ± 3.1 ng.mg^−1^ dry tissue weight, respectively, *n* = 6 ± 95% confidence level [CL])), compared to matched cellular control tissue (79.6 ± 13.9, 94.3 ± 25.1, and 291.5 ± 42.8 ng.mg^−1^ dry tissue weight, respectively, *n* = 6 ± 95% CL) (Fig. 4a, b respectively). In these tissue regions, the mean total DNA content of the decellularized tissue was below 50 ng.mg^−1^ dry weight. These reductions were found to be significant (*p* = 0.002; Mann–Whitney *U* test). The mean total DNA content of the decellularized NP region was not significantly different from the cellular control tissue (90.5 ± 18.3 and 87.7 ± 14.9 ng.mg^−1^ dry tissue weight respectively, *n* = 6 ± 95% CL) (*p* = 0.937; Mann–Whitney *U* test).

##### Glycosaminoglycan

Sulfated GAG levels for cellular NP, iAF, and oAF tissue regions were 417.4 ± 86.9, 260.4 ± 53.6, and 26.0 ± 16.5 μg.mg^−1^ dry tissue weight, respectively, *n* = 6 ± 95% CL (Fig. 3c). Values for decellularized NP, iAF, and oAF tissue regions were 421.6 ± 84.9, 225.0 ± 55.6, and 15.2 ± 4.7 μg.mg^−1^ dry tissue weight, respectively, *n* = 6 ± 95% CL (Fig. 4d). Sulfated GAG levels between cellular and decellularized NP, iAF, and oAF were not significantly different (*p* = 1.000, 0.180, and 0.093, respectively, Mann–Whitney *U* test).

#### Biomechanical evaluation of bovine specimens

**FIG. 5.**
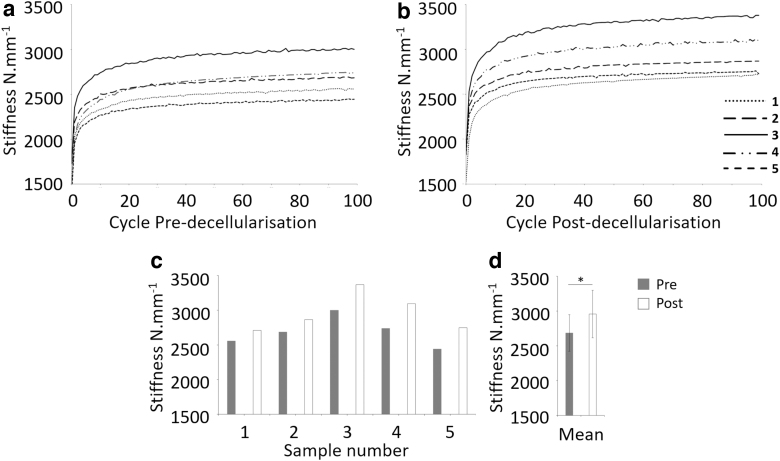
Stiffness (N.mm^−1^) of individual bovine EP-IVD-EP samples predecellularization **(a)** and postdecellularization **(b)** for 100 load-unload cycles. Mean stiffness values for individual EP-IVD-EP predecellularization and postdecellularization, calculated from stiffness values obtained from the last 20 load-unload cycles **(c)**. Mean stiffness of samples predecellularization and postdecellularization, calculated from data shown in **(c) (d)**; data are expressed as the mean (*n* = 5) ± 95% CL. *Significant difference between predecellularization and postdecellularization, *p* = 0.043 (Wilcoxon signed rank test). CL, confidence level.

The stiffness of all samples (predecellularization and postdecellularization) increased with each load-unload cycle. The increase became less pronounced after 20 load-unload cycles and reached a near-plateaux by 100 cycles (Fig. 5a, b).

The mean stiffness data from the last 20 load-unload cycles of each of the samples, predecellularization and postdecellularization, are shown in Figure 5c. The stiffness of all samples increased postdecellularization. Mean data (*n* = 5) for the last 20 load-unload cycles are shown in Figure 5d. The mean stiffness data were significantly different predecellularization and postdecellularization (*p* = 0.043; Wilcoxon signed rank test). This change equated to a mean increase in stiffness of 9.2%. The variance in the stiffness between specimens, predecellularization and postdecellularization, is shown in Figure 5c; the difference between the highest and lowest stiffness values was 20.7% predecellularization and 21.8% postdecellularization.

#### Macroscopic images of human specimens

**FIG. 6.**
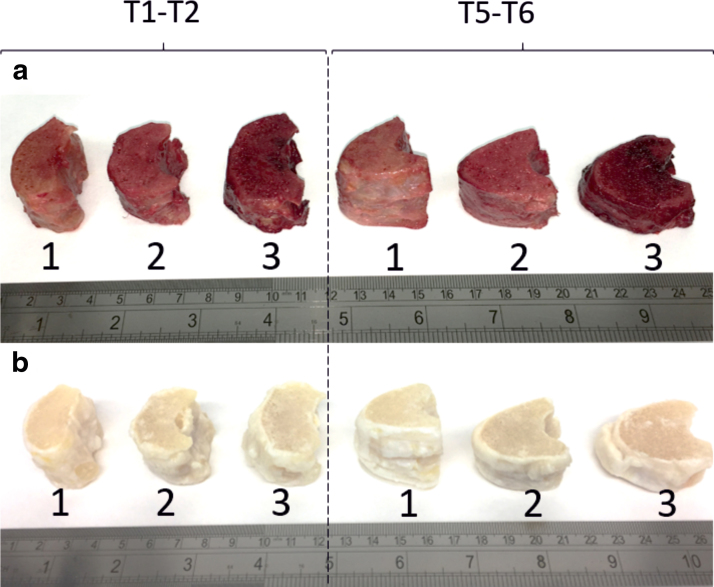
Macroscopic images of human cellular **(a)** and decellularized **(b)** VB-IVD-VB samples. Samples are from the T1–T2 and T5–T6 thoracic regions of spines from three individual donors (1, 2, and 3). Color images are available online.

Figure 6a and b show macroscopic images of VB-IVD-VB samples before and after decellularization, respectively. The images highlight the variation in size and shape of samples from the same thoracic levels taken from separate donor spines. Decellularized samples are white as a result of blood and bone marrow removal and bleaching by PAA during the decellularization process.

#### Histological evaluation of human specimens

**FIG. 7.**
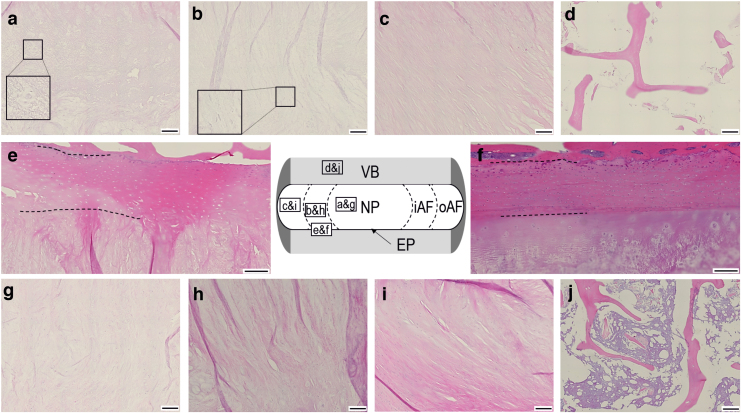
Images of H&E-stained tissue sections of human IVD tissue regions. Decellularized NP **(a)**; iAF **(b)**; oAF **(c)**; EP **(d);** and VB **(e)**. Cellular VB **(f)**; NP **(g)**; iAF **(h)**; oAF **(i);** and EP **(j)**. The central diagrammatic representation of a human IVD cross-section shows the location of the tissue regions shown in the individual images (*boxed areas* with image labels). Images **(a, b)** include magnified *inset images* of selected regions. The *dashed lines* in images e and f show the boundary of the cartilaginous EP, with the vertebral EP (*above*) and IVD enthesis (*below*). All images taken at × 200 magnification with 200 μM scale bars. Color images are available online.

**FIG. 8.**
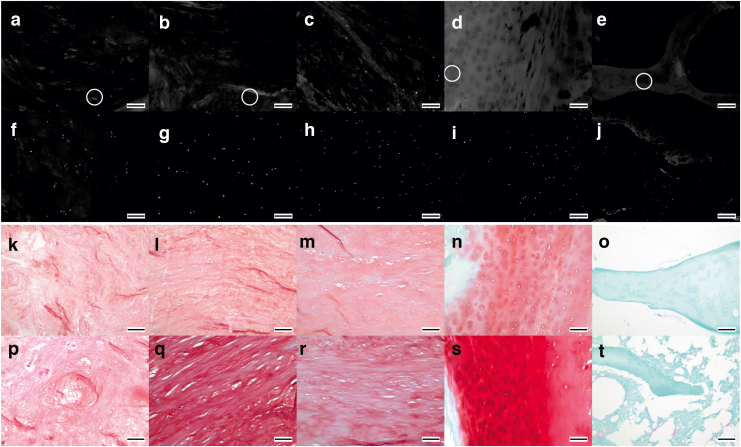
Images of histologically stained tissue sections of human IVD tissue regions. DAPI-stained sections of decellularized tissue: NP **(a)**; iAF **(b)**; oAF **(c)**; EP **(d)**; and VB **(e)** and cellular tissue: NP **(f)**; iAF **(g)**; oAF **(h)**; EP **(i)**; and VB **(j)**. Circled areas contain examples of faintly stained nuclear material. Safranin O-/fast green stained sections of decellularized tissue: NP **(k)**; iAF **(l)**; oAF **(m)**; EP **(n)**; and VB **(o)** and cellular tissue: NP **(p)**; iAF **(q)**; oAF **(r)**; EP **(s)**; and VB **(t)**. All images taken at × 100 magnification with 100 μM scale bars. Color images are available online.

The majority of decellularized tissue was devoid of cells. There was a reduction in nuclear material across all decellularized tissue regions stained with H&E and DAPI (Figs. 7a–e and 8a–e) compared to cellular tissue regions (Figs. 7f–j and 8f–j). In the decellularized NP, iAF, oAF, EP, and VB, there was no clearly defined staining for nuclear material in sections stained with H&E, suggesting that no whole nuclei were present (Fig. 7a–e). Some H&E-stained sections of NP and iAF showed “ghost nuclei” (Fig. 7a, b insets). DAPI-stained sections also showed faint nuclear material in the NP (Fig. 8a, circled) and to a lesser degree in the iAF (Fig. 8b, circled). The fluorescence intensity of the DAPI was lower in decellularized tissue sections than in cellular tissue sections.

Whole cell nuclei were observed in some areas of the oAF stained with DAPI (Fig. 7c), while other areas were completely negative. There was an occasional whole nucleus in DAPI-stained sections of decellularized VB (Fig. 7e, circled) and faint areas of nuclear material were seen in the EP (Fig. 8d, circled); these tissue regions were generally free of nuclear material. Bone marrow was largely removed and if present, appeared devoid of cell nuclei in H&E- and DAPI-stained sections (Figs. 7d and 8e).

GAGs were retained in decellularized NP, iAF, oAF, and EP. Mean (two specimens from three donors) Safranin O staining intensity scores for cellular NP, iAF, oAF, EP, and VB were 3.6, 4, 3.8, 4.5, and 0, respectively. Mean scores for decellularized NP, iAF, oAF, EP, and VB were 3.7, 3.5, 3.8, 3.7, and 0, respectively. Decellularized tissue regions (Fig. 8k–n) showed a slightly lower Safranin O staining intensity than respective cellular tissue regions (Fig. 8p–s). No Safranin O staining was observed in cellular or decellularized VB (Fig. 8, t respectively).

Tissue histoarchitecture was retained postdecellularization. ECM in decellularized NP sections (Fig. 8k) showed a random alignment, similar to cellular tissue (Fig. 8p). The parallel alignment of ECM fibers was retained in the decellularized iAF and oAF (Fig. 8l, m) when compared to the cellular iAF and oAF (Fig. 8q, r respectively). There were no apparent differences in the histoarchitecture of decellularized EP and VB (Fig. 8n, o), when compared to cellular tissue (Fig. 8s, t).

#### Biochemical evaluation of human specimens

##### DNA

**FIG. 9.**
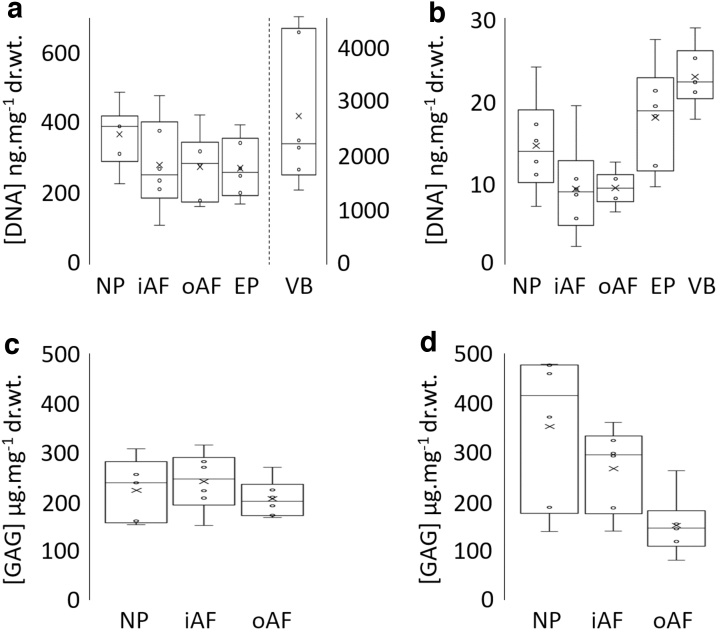
Mean (*n* = 3: six specimens) total DNA content (ng.mg^−1^ dry tissue weight) of cellular **(a)** and decellularized **(b)** human IVD tissue regions. Mean (*n* = 3: six specimens for iAF and oAF; five specimens for NP) sulfated GAG content (μg.mg^−1^ dry tissue weight) of cellular **(c)** and decellularized **(d)** human IVD tissue regions. x = mean; ○ = individual data points; mid-line of box = median; Bars = extreme values; Box = interquartile range.

Variation was seen in total DNA content of cellular tissue for each tissue region (NP: 229 to 489; iAF: 110 to 479; oAF: 164 to 424; EP: 171 to 396; and VB 1382 to 4621 ng DNA.mg^−1^ dry tissue, Fig. 9a). The total DNA content in all the decellularized tissue regions of all six decellularized IVDs was below 50 ng.mg^−1^ dry tissue (Fig. 9b). The lowest value of all decellularized tissue regions was 2 ng.mg^−1^ (iAF) and the highest was 29 ng.mg^−1^ (VB).

##### Glycosaminoglycan

Both cellular and decellularized NP, iAF, and oAF showed a large variation in sulfated GAG content (cellular NP: 153 to 307, iAF: 151 to 314, and oAF: 168 to 269 μg.mg^−1^ dry tissue weight, Fig. 9c; and decellularized NP: 139 to 477, iAF: 140 to 360, and oAF: 81 to 262 μg.mg^−1^ dry tissue weight, Fig. 9d). The GAG content profiles of five of the decellularized samples showed a decrease in GAG content from the NP to the iAF to the oAF, suggesting that GAGs were removed more readily from the outer regions of the IVD.

## Discussion

The aims of this initial proof of technical feasibility study were to develop and assess the performance of a decellularization protocol on an animal model (bovine tail IVDs with EPs), before application of the protocol to human IVDs with attached VB. It is proposed that decellularized human IVDs have the potential to be developed for future replacement of degenerate IVDs.

As IVDs are well integrated into adjacent VB through the EP, retaining these structures in the decellularized tissues would preserve the structural integrity of the IVD and provide a fixation point for implantation. Indeed, the EPs of cervical IVD allografts have been shown to integrate with recipient VB.^19,20^ There is also evidence that decellularized structural allografts of other tissues can restore function and undergo constructive remodeling *in vivo*. Decellularized allogeneic cardiac valves are used clinically,^26–29^ while decellularized functional musculoskeletal tissues have been shown to osseointegrate and undergo constructive remodeling in animal studies,^30,31^ thus supporting the concept of decellularized IVD implantation to replace degenerate IVDs.

Stringent selection criteria for donor IVDs would ensure good quality healthy tissue (IVD and EP) for decellularization to produce biological scaffold implants for the replacement of degenerate tissue in the recipient.

Studies in primates have shown that size matching of implanted allogeneic IVDs is important in preventing graft migration.^32,33^ The IVDs of the human lumbar spine, a common region for IVD degeneration, are particularly large with unique geometries and so the most likely source of replacement tissue is cadaveric human donors.^34–39^ Large bovine tail IVDs were selected for protocol development due to their size and their similar properties to human lumbar IVDs.^40,41^ This facilitated the translation of the protocol to similarly sized human thoracic IVDs, allowing the application of consistent protocol parameters (e.g., solution volumes, agitation, and treatment durations). This enabled proof of technical feasibility of the decellularization of human IVDs. Future studies to investigate decellularization of larger human lumbar IVDs, including a larger more representative sample size, are now warranted as part of the next steps toward clinical translation.

The decellularization process was adapted from the protocol developed for porcine menisci.^18^ In the current protocol, the incorporation of sonication, antibiotic, weekend-long wash steps and larger wash solution volumes was made; incubation temperatures were altered and EDTA was omitted from wash solutions.

The majority of tissue regions in bovine and human IVDs were decellularized effectively. Nuclei removal proved more difficult in two regions. Decellularized bovine NP had some intact nuclei within the high-density cellular regions and DNA content was not significantly different to native NP. It is proposed that these cell clusters were notochord-like cells and that their central location and surrounding dense matrix were a barrier to decellularization.^42^ Human notochord cells diminish after the age of 10^43^ and cell clusters were not observed in the mature human NP tissue used in this study; the human NP was effectively decellularized.

The enthesis regions of bovine tissue, NP-EP and AF-EP junctions, were also difficult to decellularize. This occurs in bone-ligament entheses, due to the dense ECM limiting the penetration of decellularization solutions.^44^ In human tissue, decellularization was variable in the oAF enthesis region only, perhaps as a result of more fibrous tissues in aging IVDs.^45,46^

Three human spines were investigated in this initial study (two IVDs from each spine). Statistical analysis was not carried out on quantitative data since there were only three true replicates. DNA quantitation, however, showed that decellularization reduced *total* DNA content in all tissue regions of the six IVDs to 2–29 ng.mg^−1^ dry tissue weight, well below the recommended target of 50 ng of double-stranded DNA per mg dry tissue.^8^

The presence of an occasional cell nucleus in the decellularized VB is unlikely to be detrimental, since washed allogeneic bone grafts, containing dead donor cells, are routinely used in orthopedic surgery.^47–51^ Decellularized bone with minimal cell remnants may osseointegrate faster than cellular allograft, as macrophages would have fewer cells to clear before bone remodelling.^49^

GAGs are present in large amounts in the IVD NP where they attract and retain water and play an important role in compressive function.^2,3,52^ GAGs were largely preserved in bovine and human IVDs postdecellularization. Minor reductions occurred in the NP with the greatest reductions in the iAF and oAF.

To understand the effects of decellularization on IVD biomechanical properties, biomechanical tests were carried out on the same bovine IVDs before and after decellularization. The tests were designed to examine the functionality of the IVD over multiple loading cycles to detect the biomechanical effect of any change in the GAG retention and ability of the disc to attract and retain water. The standardization of test conditions predecellularization and postdecellularization allowed for robust comparisons and was sufficiently sensitive to detect a small increase in IVD stiffness (9.2%) postdecellularization. This change was within the variation of stiffness observed in both untreated and decellularized specimens (20.7% and 21.8%, respectively).

These changes were most likely due to the small decrease in GAG content in the decellularized tissue. In studies of GAG-depleted bovine IVDs in which the NP was enzymatically digested, the stiffness of the IVDs increased substantially post-treatment (mean 25% ± 10% standard deviation [SD]).^53,54^ GAG loss in human degenerate IVDs results in a loss of disc height, a compacted structure, and a consequent increase in compressive modulus.^55,56^ If the decellularized IVDs were less swollen due to decreased hydration, they would have a more compact structure and the applied load would have been shifted onto the collagen component (in particular the collagen-rich AF).^55,57^ Other studies where decellularization of musculoskeletal tissue was carried out using 0.1% (w/v) SDS also showed GAG reductions with minimal changes to the mechanical properties.^18,58–61^

Further investigations into the effects of the decellularization process on the biocompatibility, biomechanics, and regenerative potential of increased numbers of human IVDs is now warranted using protocols established in this and previous studies by our group. These investigations should include the following: biomechanical analysis of decellularized human IVD predecellularization and postdecellularization as described in this study for bovine IVDs; assessment of decellularized tissue biocompatibility *in vitro* and *in vivo*^62^; determination of residual SDS content^62^ and residual DNA fragment size^18^; and immunohistochemical analysis of matrix components.^63^ There is also scope to couple the decellularized scaffold with autologous minimally manipulated multipotential stromal cells in future applications.

## Conclusion

This study provides evidence that decellularization of whole IVDs is possible. There is potential for the use of decellularized human IVDs as a replacement for degenerate IVDs. Further investigations are required before this technology can be taken forward to clinical application. To our knowledge, this is the first study to decellularize large IVDs with VB attachments for the ultimate purpose of producing an implant for the replacement of degenerate IVDs. It is also the first to apply decellularization protocols to human IVDs.
